# Supplementary material for: Fibrous Materials Made of Poly(ε-caprolactone)/Poly(ethylene oxide)-b-Poly(ε-caprolactone) Blends Support Neural Stem Cells Differentiation
Source: Polymers (Basel). 2019 Oct 8;11(10):1621. doi: 10.3390/polym11101621 (PMC6835932; doi:10.3390/polym11101621)
Supplement: Supplementary file 1 [file polymers-11-01621-s001.pdf]

polymers

Supplementary Material for the article:

Fibrous Materials Made of Poly(ε-caprolactone)/Poly(ethylene oxide)-*b*-Poly(ε-caprolactone) Blends Support Neural Stem Cells Differentiation

1. Block Copolymers Characterization

1.1. Structure and Molecular Weight by ^{1}H-NMR and GPC

The block copolymers have been characterized by proton nuclear magnetic resonance (^{1}H-NMR, Bruker, 600 MHz) and gel permeation chromatography (GPC, Jasco). Samples for ^{1}H-NMR characterization were dissolved in deuterated chloroform, and samples for GPC were dissolved in THF. Figure S1 shows the corresponding ^{1}H-NMR spectra of the PEO_m macroinitiator and the resulting PEO_m-*b*-PCL_n block copolymers. For the macroinitiator, the protons corresponding to the repeating unit of ethylene oxide moiety (H_b′) are observed at 3.67 ppm as a singlet, meanwhile the methoxy terminal protons (H_a′) appear at 3.41 ppm as a singlet, and the terminal hydroxyl proton (H_c′) appear at 2.16 ppm also as a singlet. The chemical shift corresponding to H_b′ protons in the synthesized block copolymers are coincident with those of the macroinitiator; however, the corresponding resonance peaks of H_a′ and H_c′ are lost in the block copolymer spectrum, due to the low intensity of these resonance peaks. Protons H_g, H_c, $H_{d–f}$, and H_e of ε-caprolactone appear at 4.06 (t), 2.31 (t), 1.65 (m), and 1.38 (m) ppm, respectively.

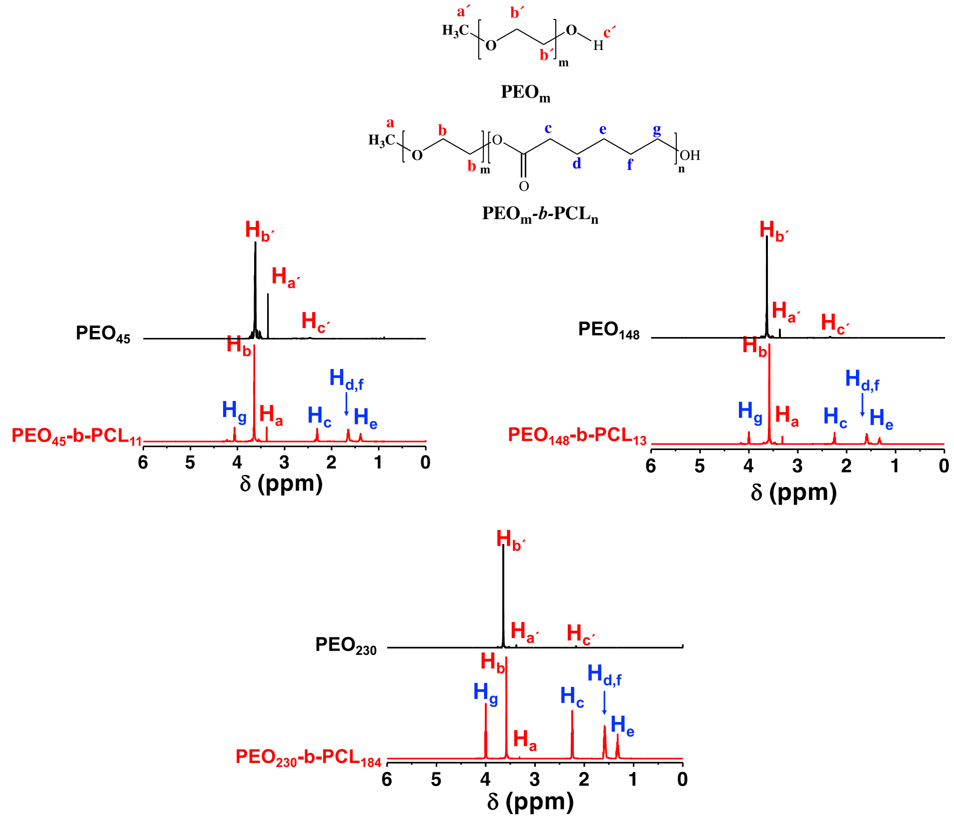

Figure S1. Proton nuclear magnetic resonance (^{1}H-NMR) spectra corresponding to the macroinitiators PEO_m, and the corresponding of diblock copolymers.

The degree of polymerization of PEO (averaged number of repeating units, N_{PEO}) can be calculated comparing the intensities of the ^{1}H-NMR resonance peaks of methyl end-group and the methylene groups. The results show good agreement with those reported by the supplier. Our own results are used in this work. Once N_{PEO} is known, similar magnitude for PCL (N_{PCL}) can be calculated using an integral method of ^{1}H-NMR resonance peaks [3], by comparing proton peak intensities of the PEO methylene repeating units (H_b) and that corresponding to, for example, proton H_c of PCL. The area or intensity of a proton resonance peak of a given species is proportional to the amount of these species present in the sample, and thus:

$$A_{H_b} = k4N_{PEO} \quad (1)$$

where k is a proportionality constant, and

$$A_{H_c} = k2N_{PCL} \quad (2)$$

Rearranging,

$$N_{PCL} = \frac{2N_{PEO}A_{H_c}}{A_{H_b}} \quad (3)$$

M_n of the copolymers can be calculated as:

$$M_n = N_{PEO}M_{\mathrm{EO}} + N_{PCL}M_{\mathrm{CL}} + M_e \quad (4)$$

where M_{EO} is the molecular weight of ethylene oxide, M_{CL} is the molecular weight of number of *ε*-caprolactone, and M_e corresponds to the molecular weight of the PEO methyl end-groups. Data corresponding to the analysis of molecular weight of the block copolymers and polydispersity index (PDI) are shown in Table S1. For comparison, molecular weight measurements by GPC (THF as solvent), using narrow molecular weight PEO as standards, are also shown in Table S1.

Table S1. Molecular weight estimation of PEO_m and PEO_m-*b*-PCL_n block copolymers by integral ^{1}H-NMR calculations and by gel permeation chromatography (GPC) measurements.

Sample	M / I	N_{PEO}	N_{PCL}	M_n ^{1}H-NMR	M_n GPC	M_w GPC	PDI
PEO_{45}	-	45	-	2,027	1,607	2,175	1.35
PEO_{45}-*b*-PCL_{11}	10.84	45	11	3,415	1,918	3,007	1.57
PEO_{148}	-	148	-	6,531	3,585	5,789	1.61
PEO_{148}-*b*-PCL_{13}	10.84	148	13	8,259	3,035	6,366	2.10
PEO_{230}	-	230	-	10,165	5,954	10,362	1.74
PEO_{230}-*b*-PCL_{184}	180	230	184	34,031	14,261	23,460	1.64

2. Internal Structure of PCL Fibers.

2.1. X-Ray Diffraction (XRD) and Small-Angle X-Ray Scattering (SAXS)

The internal crystalline structure of PCL fibrous materials has been characterized by XRD and SAXS. Figure S2 shows the x-ray diffractogram of the fibrous materials. Two peaks appearing at 2θ of 21.98 and 24.22°, related with the (110) and (200) crystallographic planes of PCL are observed, characteristics of the orthorhombic lattice structure of PCL.

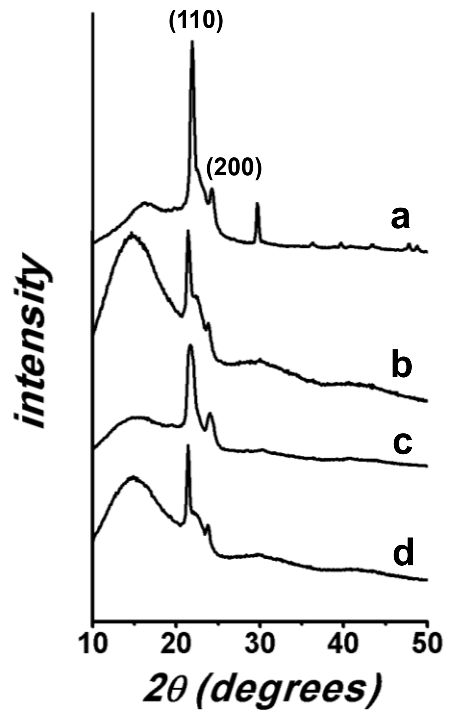

Figure S2. XRD diffractogram of fibers prepared using PCL_{394} (**a**), PCL_{394}/PEO_{45}-*b*-PCL_{11} (**b**), PCL_{394}/PEO_{148}-*b*-PCL_{13} (**c**), and PCL_{394}/PEO_{230}-*b*-PCL_{184} (**d**).

The Scherrer equation was used to estimate the thickness of the crystallite perpendicular to the lattice plane (L), according to:

$$L = \frac{K\lambda}{\beta cos\theta} \quad (5)$$

where K correspond to a shape factor with a value of 1, λ represent the X-ray wavelength from monochromatic Cu K-α radiation (0.154184 Å), β corresponds to the half at the maximum intensity (FWHM) of peaks observed in diffractogram, and θ is the Bragg angle. Pristine PCL_{394} fibers present crystalline domains of 17 nm, meanwhile in those prepared using block copolymers of PEO_{45}-*b*-PCL_{11} and PEO_{230}-*b*-PCL_{184}, the ordered domain increases from 19–21 nm to 22–26 nm, respectively, and a decrease to 9 nm in the presence of PEO_{148}-*b*-PCL_{13} is observed.

2.2 Small-Angle X-Ray Scattering (SAXS)

Complementary to XRD, SAXS measurements where done in order to study the changes in the crystalline/amorphous domains of PCL_{394} fibers in the presence of block copolymers. In the Figure S3 it is shown the one-dimensional SAXS plots (1D-SAXS) of fibrous materials, where a clear peak centered around 0.50 nm^{-1} (q_{max}) is observed, characteristic of a lamellar morphology [7–9]. This peak is a result of the electron density differences between the crystalline and amorphous domains of PCL in the fibers [8]. The long period (L') of semicrystalline PCL structure, corresponding to the periodicity of lamellar stacks, can be estimated from SAXS curves using the equation:

$$L' = \frac{2\pi}{q_{max}} \quad (6)$$

In the fibrous materials prepared using PCL_{394}, the long period is approximately 12–13 nm. The peak observed in 1D-SAXS plots does not shift at higher or lower q in the presence of the block copolymers. However, an increase on the intensity in the low-q range is observed, due to fluctuations

on the electronic density, indicating a relative loss of crystallinity. The absence of high order peaks in the 1D-SAXS is an indicative of the lack of other lamellar superstructures.

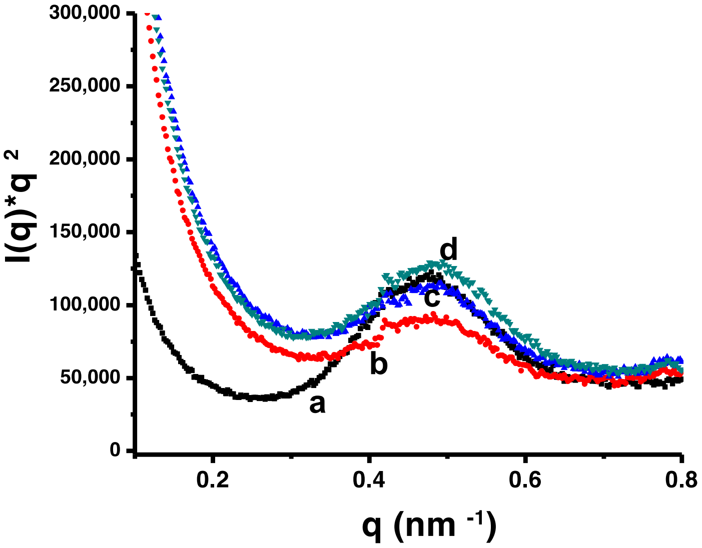

Figure S3. Kratky small-angle X-ray scattering (SAXS) plot (represented as $I(q){\cdot}q^2$ versus q) for PCL_{394} (**a**), PCL_{394}/PEO_{45}-*b*-PCL_{11} (**b**), PCL_{394}/PEO_{148}-*b*-PCL_{13} (**c**), and PCL_{394}/PEO_{230}-*b*-PCL_{184} (**d**).

In order to obtain a detailed description about changes in the lamellar packing of PCL_{394} in the long-range order, in the absence and in the presence of the block copolymers, the one-dimensional correlation functions were computed using the cosine transformation from scattering intensity curves using:

$$K(r) = \frac{1}{Q}\int_0^\infty I(q)q^2\cos(rq)\,dq \tag{7}$$

Where r represents the distance in real space, and Q correspond to the invariant function, defined as:

$$Q = \int_0^\infty I(Q)Q^2 dQ \tag{8}$$

As observed in Figure S4, the structural parameters such as the long period (L_p) and the lamellar thickness (L_c) are obtained from the first local minima ($K(r) < 0$) and the first local maxima (with $K(r)$ > 1).

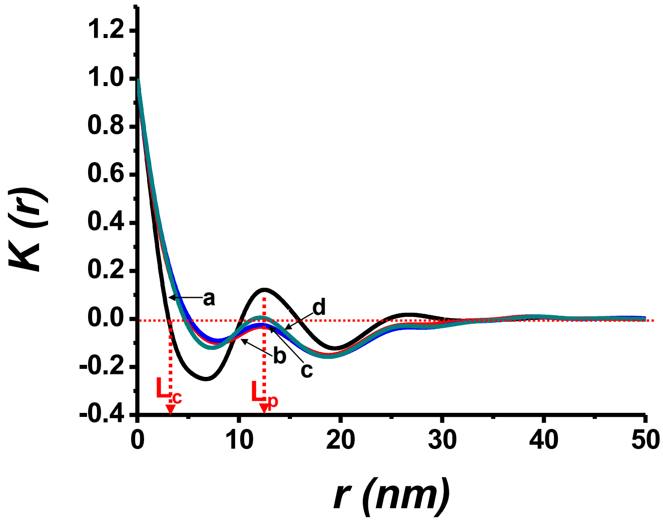

Figure S4. Normalized one-dimensional correlation functions, *K*(*r*), for PCL_{394} (**a**), PCL_{394}/PEO_{45}-*b*-PCL_{11} (**b**), PCL_{394}/PEO_{148}-*b*-PCL_{13} (**c**), and PCL_{394}/PEO_{230}-*b*-PCL_{184} (**d**).

3. Thermal Analysis

3.1. Differential Scanning Calorimetry (DSC)

DSC measurements where done to finely characterize the melting and crystallization temperature of PCL_{394} in the absence and in the presence of the block copolymers, allowing calculation of the crystallinity ratio from enthalpies of melting state, by comparing with the enthalpy of melting of 100% crystalline PCL (139.5 J/g). DSC heating and cooling cycles are shown in the Figure S5. The presence of the block copolymers in the fibers does not produce significant changes in the position of the melting and the crystallization temperatures. However, differences in the enthalpy of melting of bulk PCL_{394} and PCL_{394} fibrous materials are observed, suggesting that the processing of bulk PCL_{394} into fibrous materials or in the presence of the block copolymers changes the crystallinity ratio. The blending of PCL with the block copolymers produces a reduction in the crystallinity ratio of the main component, achieving a 45% of crystallinity in materials prepared with the block copolymer PEO_{45}-*b*-PCL_{11}. Similar crystallinity ratios were obtained in PCL_{394} blended with PEO_{148}-*b*-PCL_{13} and PEO_{230}-*b*-PCL_{184}.

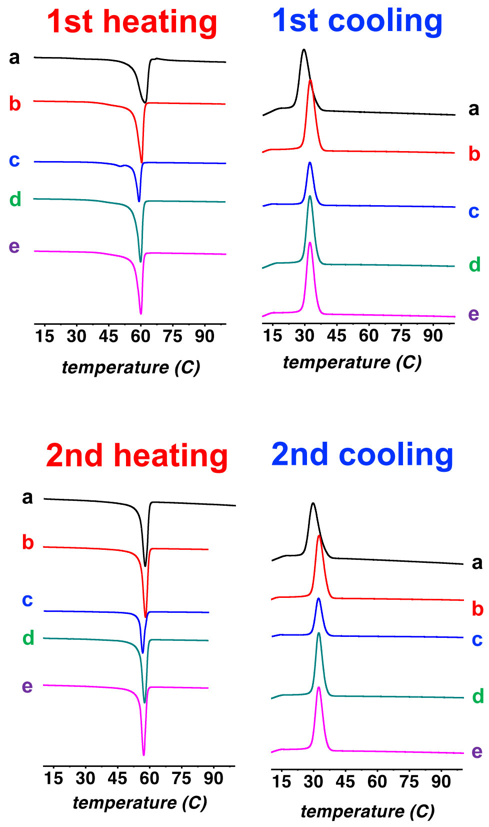

Figure S5. DSC curves corresponding to bulk PCL_{394} (**a**), and fibers composed by pristine PCL_{394} (**b**), and those blended with the block copolymers PEO_{45}-*b*-PCL_{11} (**c**), PEO_{148}-*b*-PCL_{13} (**d**), and PEO_{230}-*b*-PCL_{184} (e), respectively.

3.2. Dynamical Mechanical Analysis (DMA)

PCL is a semicrystalline polymer with characteristic transition temperatures such as the glass transition (T_g) and melting point (T_m). Although DSC is a powerful technique to characterize T_m, it loses sensibility to characterize T_g [10,11]. We performed DMA measurement to characterize the glass transition of PCL_{394} in order to evaluate the effect of the block copolymers in these phase transitions. The results are shown in Figure S6 as storage modulus and *tan δ* as a function of temperature. Although high values of storage modulus are observed, these are related with the use of the metallic material pocket where the polymer samples were loaded for DMA measurement. The storage modulus of the studied samples decreased at around −50 °C, associated to the glass transition of PCL. As long as the temperature increases, a second pronounced decrease on the storage modulus is observed around 50 °C, assigned to the melting of PCL. Small shifts of the maximum of tan δ to lower and higher temperatures are observed when pristine PCL fibers are compared with those prepared using block copolymers.

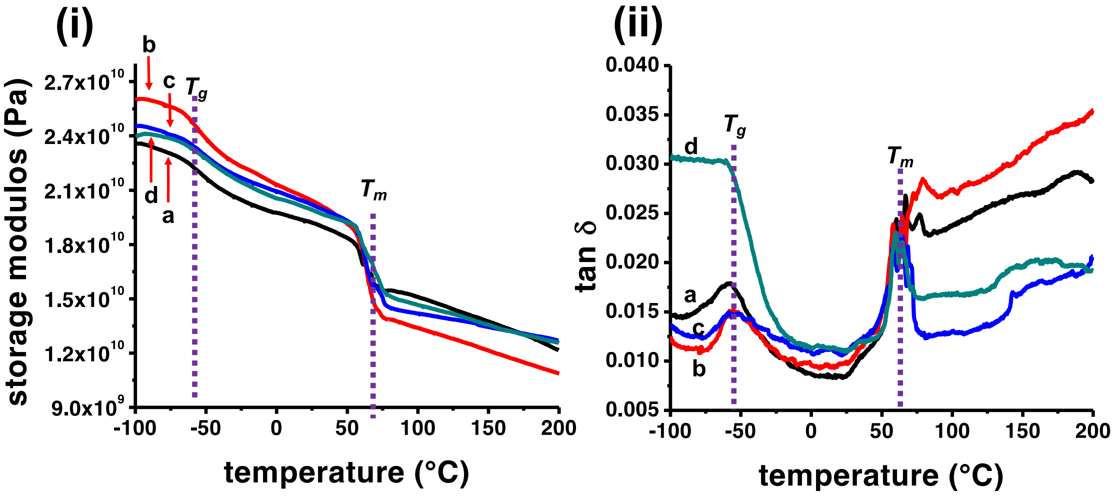

Figure S6. Storage modulus and *tan δ* as function of temperature of PCL_{394} (a), PCL_{394}/PEO_{45}-*b*-PCL_{11} (b), PCL_{394}/PEO_{148}-*b*-PCL_{13} (c), and PCL_{394}/PEO_{230}-*b*-PCL_{184} (d).

References

1. Meier, M.A.R.; Aerts, S.N.H.; Staal, B.B.P.; Rasa, M.; Schubert, U.S. PEO-b-PCL Block Copolymers: Synthesis, Detailed Characterization, and Selected Micellar Drug Encapsulation Behavior. *Macromol. Rapid Commun.* **2005**, *26*, 1918–1924. doi:10.1002/marc.200500591.
2. Flores, M.E.; Martínez, F.; Olea, A.F.; Shibue, T.; Sugimura, N.; Nishide, H.; Moreno-Villoslada, I. Stability of Water/Poly(ethylene oxide)43-b-poly(ε-caprolactone)14/Cyclohexanone Emulsions Involves Water Exchange between the Core and the Bulk. *J. Phys. Chem. B* **2015**, *119*, 15929–15937. doi:10.1021/acs.jpcb.5b10274.
3. Izunobi, J.U.; Higginbotham, C.L. Polymer Molecular Weight Analysis by 1H NMR Spectroscopy. *J. Chem. Educ.* **2011**, *88*, 1098–1104. doi:10.1021/ed100461v.
4. Hu, H.; Dorset, D.L. Crystal structure of poly(ε-caprolactone). *Macromolecules* **1990**, *23*, 4604–4607. doi:10.1021/ma00223a017.
5. Ajili, S.H.; Ebrahimi, N.G. Miscibility of TPU(PCL diol)/PCL Blend and Its Effect on PCL Crystallinity. *Macromol. Symp.* **2007**, *249-250*, 623-627, doi:10.1002/masy.200750446.
6. Mirhosseini, M.M.; Haddadi-Asl, V.; Zargarian, S.S. Fabrication and characterization of hydrophilic poly(ε-caprolactone)/pluronic P123 electrospun fibers. *J. Appl. Polym. Sci.* **2016**, *133*. doi:10.1002/app.43345.
7. Ryan, A.J.; Bras, W.; Mant, G.R.; Derbyshire, G.E. A direct method to determine the degree of crystallinity and lamellar thickness of polymers: Application to polyethylene. *Polymer* **1994**, *35*, 4537–4544. doi:10.1016/0032-3861(94)90799-4.
8. Kim, J.K.; Park, D.-J.; Lee, M.-S.; Ihn, K.J. Synthesis and crystallization behavior of poly(l-lactide)-block-poly(ϵ-caprolactone) copolymer. *Polymer* **2001**, *42*, 7429–7441. doi:10.1016/S0032-3861(01)00217-8.
9. Germiniani, L.G.L.; da Silva, L.C.E.; Plivelic, T.S.; Gonçalves, M.C. Poly(ε-caprolactone)/cellulose nanocrystal nanocomposite mechanical reinforcement and morphology: The role of nanocrystal pre-dispersion. *J.Mater. Sci.* **2019**, *54*, 414–426. doi:10.1007/s10853-018-2860-9.
10. Achorn, P.J.; Ferrillo, R.G. Comparison of thermal techniques for glass transition measurements of polystyrene and cross-linked acrylic polyurethane films. *J. Appl. Polym. Sci.* **1994**, *54*, 2033–2043. doi:10.1002/app.1994.070541305.
11. Abiad, M.G.; Campanella, O.H.; Carvajal, M.T. Assessment of Thermal Transitions by Dynamic Mechanical Analysis (DMA) Using a Novel Disposable Powder Holder. *Pharmaceutics* **2010**, *2*, 78–90. doi:10.3390/pharmaceutics2020078.

© 2019 by the authors. Submitted for possible open access publication under the terms and conditions of the Creative Commons Attribution (CC BY) license (http://creativecommons.org/licenses/by/4.0/).
